# Modulation of Dormancy and Growth Responses in Reproductive Buds of Temperate Trees

**DOI:** 10.3389/fpls.2018.01368

**Published:** 2018-09-13

**Authors:** Alba Lloret, María Luisa Badenes, Gabino Ríos

**Affiliations:** Centre of Citriculture and Plant Production, Instituto Valenciano de Investigaciones Agrarias (IVIA), Valencia, Spain

**Keywords:** bud dormancy, cold acclimation, epigenetic regulation, flowering, peach (*Prunus persica*)

## Abstract

During autumn perennial trees cease growth and form structures called buds in order to protect meristems from the unfavorable environmental conditions, including low temperature and desiccation. In addition to increased tolerance to these abiotic stresses, reproductive buds modulate developmental programs leading to dormancy induction to avoid premature growth resumption, and flowering pathways. Stress tolerance, dormancy, and flowering processes are thus physically and temporarily restricted to a bud, and consequently forced to interact at the regulatory level. We review recent genomic, genetic, and molecular contributions to the knowledge of these three processes in trees, highlighting the role of epigenetic modifications, phytohormones, and common regulatory factors. Finally, we emphasize the utility of transcriptomic approaches for the identification of key structural and regulatory genes involved in bud processes, illustrated with our own experience using peach as a model.

## Life in a Bud

Boreal and temperate tree species cope with harsh environmental conditions during autumn and winter, including low and freezing temperatures, in a quiescent state named dormancy. Dormancy has been originally defined as the absence of visible growth in a meristematic structure. Traditionally, three types of dormancy have been distinguished according to physiological cues leading to growth inhibition: endodormancy, when signals are intrinsic to the meristem; paradormancy, imposed by another part of the plant; and ecodormancy, due to environmental factors (Lang, 1987). More recently, dormancy has been reformulated as “the inability to initiate growth from meristems under favorable conditions” (Singh et al., 2017). When applied to buds, this general definition covers endodormancy, and axillary bud paradormancy imposed by apical dominance, but not bud growth inhibition by environmental factors after fulfillment of chilling requirements (ecodormancy). In this review, we use the term dormancy referring to this second meaning.

Previously, prominent reviews have also addressed the known mechanisms of bud dormancy control in perennial plants from a molecular perspective (Rohde and Bhalerao, 2007; Allona et al., 2008; Anderson et al., 2010; Yamane, 2014; Maurya and Bhalerao, 2017; Singh et al., 2017). Several of these studies have focused on the molecular control of growth arrest in apical vegetative meristems. Growth cessation and dormancy induction in those meristems are regulated by endogenous and environmental signals, being photoperiod shortening and temperature lowering major determinants of dormancy setup in forest species and Rosaceae fruit trees, respectively (Heide and Prestrud, 2005; Cooke et al., 2012). By contrast, the development of flower lateral meristems is usually determined by apical dominance and other factors to overwinter in a differentiated immature stage, preceding dormancy release, and growth resumption on next season.

Even though diverse anatomical and physiological particularities are found, differentiated vegetative and reproductive meristems cease growth in a well defined stage and form a bud surrounded by protective scales in a similar fashion in different tree species. However, in spite of growth cessation and dormancy, overwintering buds do not remain in a completely inactive state, as verified by extensive transcriptomic and hormonal changes observed during bud development (Howe et al., 2015; Chao et al., 2017). In fact, three major processes including *dormancy, cold acclimation*, and *flowering* converge spatially and temporally in a reproductive bud, where they play an active and relevant role in bud dynamics and determine plant survival and growth resumption under favorable conditions. These processes determine bud phenology and development through their reciprocal interaction, integrating temperature, and photoperiod effects (Kurokura et al., 2013; Vitasse et al., 2014; Singh et al., 2017). Since flowering development requires cell division and expansion, and dormancy and cold acclimation are essentially non-growing processes, the control of cell cycle and growth is expected to influence the succession of the mutually incompatible periods of flowering and dormancy (Horvath et al., 2003).

The transition to reproductive growth starts around early spring in *Populus* (Boes and Strauss, 1994) and summer in many Rosaceae (Kurokura et al., 2013) when flower bud induction occurs in axillary meristems. Then flower organ differentiation starts and is substantially accomplished before dormancy initiates. Growth arrest and seasonal dormancy are induced specifically by either photoperiod or temperature in different species (Cooke et al., 2012). In parallel, cold, freezing, and desiccation tolerances are increased by an acclimation mechanism (Welling and Palva, 2006). Subsequent production of reproductive gametes and resumption of flower organ growth requires dormancy release triggered by the quantitative perception of chilling accumulated during the dormancy period (Coville, 1920; Couvillon and Erez, 1985). After dormancy release, buds remain cold-acclimated until a period of warm temperatures results in deacclimation and bud break (Welling and Palva, 2006). The whole succession of events from flower bud induction to blooming can be interpreted as a trade-off between defense factors leading to cold acclimation and dormancy and growth factors leading to dormancy release and flowering. In this context, photoperiod and temperature are important environmental inputs acting occasionally on opposing ways.

A more detailed review of recent progresses in the regulation of dormancy, cold acclimation and flowering processes in buds of temperate trees is shown below, with dormancy and growth promoting pathways addressed in separated sections. In addition, the utility of genomic approaches for the identification of genes related to these processes is illustrated with our own transcriptomic studies performed in flower buds of peach (*Prunus persica*) across dormancy release.

## Dormancy Setup

### Environmental Signals Leading to Growth Cessation

Reduction in daylength below a critical value induces growth cessation in several trees through the participation of orthologs of well known elements of the photoperiodic control of flowering in *Arabidopsis*, particularly photoreceptors and the circadian clock. One of them is *phytochrome A* (*PHYA*) gene that codes for a red/far-red light receptor and causes, respectively, impaired and faster growth cessation responses to short-days in hybrid aspen (*Populus tremula* × *Populus tremuloides*) plants overexpressing and down-regulating the gene (Olsen et al., 1997; Kozarewa et al., 2010). In addition, antisense inhibition of *PHYA* alters the expression of clock components, suggesting a link with circadian rhythms. This has been confirmed by down-regulating the circadian clock genes *LATE ELONGATED HYPOCOTYL1* (*PttLHY1*), *PttLHY2*, and *TIMING OF CAB EXPRESSION 1* (*PttTOC1*) in hybrid aspen, leading to a reduced critical daylength required for growth cessation and additional effects on winter hardiness and bud burst (Ibáñez et al., 2010). The regulatory module *CONSTANS* (*CO*)/*FLOWERING LOCUS T* (*FT*), which mediates the effect of photoreceptors and circadian clock on flowering initiation in *Arabidopsis* (Valverde et al., 2004), has been also proposed to control growth cessation and bud set in *Populus* trees (Böhlenius et al., 2006). In fact, two paralogs of *FT* act coordinately to determine vegetative and reproductive growth (Hsu et al., 2011); whereas *FT1* induces the reproductive onset, *FT2* promotes vegetative growth and inhibits bud formation under warm temperature and long photoperiod conditions. Also the *FT*/*TERMINAL FLOWER1-Like2* (*PaFTL2*) gene induces bud set under transgenic inducible expression in the conifer Norway spruce (*Picea abies*), resembling the *FT*-antagonistic role of *TERMINAL FLOWER1* (*TFL1*) in *Arabidopsis* (Karlgren et al., 2013). Finally, further evidences of the functional diversification of *CO*/*FT* module in flowering and dormancy processes arise from the study of other components of the pathway: the FT protein interactor gene *FLOWERING LOCUS D* is involved in growth cessation and bud formation (Tylewicz et al., 2015; Parmentier-Line and Coleman, 2016); and both overexpression and RNAi studies show that a tree ortholog of the flowering pathway integrator *APETALA1* (*AP1*) mediates photoperiod-dependent growth cessation in hybrid aspen (Azeez et al., 2014).

Besides photoperiod, temperature also affects seasonal growth arrest in many species and ecotypes, although signaling pathways mediating temperature-dependent effects are far less known (Heide and Prestrud, 2005; Heide, 2011; Rohde et al., 2011; Cooke et al., 2012). According to Tanino et al. (2010), the existence of independent short photoperiod and low temperature pathways for growth cessation and dormancy induction ensures a higher plasticity for adaptation to changing conditions. *DORMANCY-ASSOCIATED MADS-BOX* (*DAM*) genes are known regulators of growth cessation and dormancy induction in many perennial species (Bielenberg et al., 2008; Horvath et al., 2010; Sasaki et al., 2011; Niu et al., 2016; Wu et al., 2017a). Low temperature has been proposed to activate *DAM* promoters for dormancy induction by direct binding of the cold-dependent C-Repeat Binding Factor (CBF), as confirmed by yeast one-hybrid and transient expression experiments in pear and Japanese apricot (Saito et al., 2015; Niu et al., 2016; Zhao et al., 2018). *DAM* genes, in turn, have been proposed to directly repress *FT* in pear (*Pyrus pyrifolia*) and leafy spurge (Hao et al., 2015; Niu et al., 2016) and activate a 9-*cis*-epoxycarotenoid dioxygenase gene (*PpNCED3*) in pear (Tuan et al., 2017), providing specific mechanisms for growth inhibition and abscisic acid (ABA) accumulation in dormant buds. Low temperature mediates a transient increase in ABA content in a photoperiod-independent manner (Welling et al., 2002), however, ABA has been shown to affect dormancy induction instead of growth arrest in hybrid aspen (Tylewicz et al., 2018). Interestingly, winter temperatures and even short treatments at 4°C disrupt the circadian oscillations of *CsTOC1* and *CsLHY* expression in chestnut (*Castanea sativa*) (Ramos et al., 2005), and chilling treatments alter the expression pattern of *GIGANTEA* (*GI*) in almond, a known mediator of circadian effects on flowering in *Arabidopsis* (Barros et al., 2017). Recently, *GI* has been postulated to regulate photoperiod-dependent growth cessation in *Populus* through the activation of *FT2*, in a protein complex with *Flavin-binding, Kelch repeat, F-BOX 1* (*FKF1*), and *CYCLING DOF FACTOR* (*CDF*) gene products (Ding et al., 2018). These and other studies suggest that temperature signals converge with photoperiod on circadian clock elements to modulate seasonal growth cessation (Cooke et al., 2012), although additional effects of temperature on carbon metabolism and hormone signaling have been also proposed to contribute to growth arrest (Wingler, 2015).

### Cold Acclimation

Overwintering buds must deal with low and freezing temperatures leading to different forms of physiological and cellular injury. In addition to physical damage caused by ice nucleation and propagation, a dehydration stress is induced by changes in water potential due to the formation of extracellular ice, and the water loss inherent to bud dormancy progress. Plants may actively enhance their tolerance to low temperatures and desiccation via gene expression modification by a cold acclimation process (Wisniewski et al., 2003). Several reviews describe in detail the molecular and genetic control of cold acclimation in trees (Welling and Palva, 2006; Preston and Sandve, 2013; Fennell, 2014; Wisniewski et al., 2014), which is broadly similar to cold acclimation mechanisms reported in herbaceous plants (Thomashow, 1999; Thomashow, 2010; Knight and Knight, 2012).

Seasonal cold acclimation and bud dormancy are related processes since both are induced by similar low temperature and photoperiod conditions (Welling et al., 2002), and both are incompatible with active plant growth, which suggests the presence of common regulatory mechanisms. In fact, impairment of the photoperiodic response by overexpression of *PHYA* and down-regulation of clock *LHY* genes reduces the critical daylength for growth cessation and also prevents cold acclimation in hybrid aspen (Olsen et al., 1997; Ibáñez et al., 2010). In addition, the effect of temperature on seasonal growth cessation and cold acclimation invoke the same cold responsive (COR) pathway (Wingler, 2015). However, cold deacclimation and bud dormancy release are not concurrent events; winter buds remain cold-acclimated after dormancy release under appropriate low temperature conditions as long as meristem growth is not resumed, after which deacclimation is not any longer reversible (Kalberer et al., 2006).

In *Arabidopsis*, MYC transcription factors encoded by *INDUCER OF CBF EXPRESSION 1-2* (*ICE1-2*) are activated by specific cold-dependent post-translational modifications, causing up-regulation of *CBF1-3* genes. Subsequently, CBFs regulate most of cold responsive targets by binding to the C-repeat/drought-responsive element (CRT/DRE) (Knight and Knight, 2012). Although COR pathway has been essentially described in *Arabidopsis*, COR components and functions are conserved in perennials (Fennell, 2014; Wingler, 2015). The ectopic expression of *Arabidopsis CBF1* increases freezing tolerance in poplar and induces transcriptomic changes overlapping with *Arabidopsis* COR regulon (Benedict et al., 2006). On the other side, constitutive expression of birch *BpCBF1* increases freezing tolerance and induces known targets of *CBF* genes in *Arabidopsis* (Welling and Palva, 2008). Moreover, the ectopic expression of a peach *CBF* gene in apple induces short-day dependent dormancy, improves freezing tolerance, and delays bud break in field studies (Wisniewski et al., 2011; Artlip et al., 2014). Interestingly, this apple line overexpressing peach *CBF* causes an altered expression of *DAM*-like and *EBB*-like genes in buds, providing an explanation for its prolonged dormancy period through the regulation of key transcription factors involved in dormancy regulation (Wisniewski et al., 2015).

*CBF*-dependent cold acclimation response includes synthesis of chaperones, dehydrins, and other protective proteins, change in lipid composition of membranes, alteration of sugars metabolism, and production of storage and antioxidant compounds, among other responses aiming at alleviate cold, drought, and oxidative stresses (Welling and Palva, 2006). Dehydrins are abundant cold-responsive proteins belonging to the late embryogenesis abundant (LEA) family that have been proposed to protect cell structures and enzymes against freezing and dehydration (Graether and Boddington, 2014). Seasonal up-regulation of a dehydrin gene in bark tissue is lower and restricted to a shorter period in the *evergrowing* (*evg*) mutant of peach having a deletion in *DAM* genes, in concordance with its lower cold tolerance (Arora et al., 1992; Arora and Wisniewski, 1994; Artlip et al., 1997). Diverse chitinases have been also suggested to act as antifreeze, storage, and defense proteins induced during the transition to dormancy in spruce (González et al., 2015).

Soluble sugars and other compounds potentially able to act as compatible solutes accumulate in dormant tissues in order to confer tolerance to cold and desiccation stresses. Low temperature up-regulates *DUAL SPECIFICITY PROTEIN PHOSPHATASE 4* (*DSP4*), most likely involved in starch dephosphorylation and degradation, to increase the synthesis of oligosaccharides during winter dormancy in chestnut (Berrocal-Lobo et al., 2011). Raffinose family oligosaccharides (RFOs) including raffinose and stachyose are compatible solutes synthesized in seeds and plant tissues undergoing abiotic stresses (Sengupta et al., 2015). Genes coding for the enzyme galactinol synthase (GolS) catalyzing the first step in the synthesis of RFOs are up-regulated in dormant buds and other tissues of woody perennials (Ko et al., 2011; Ibáñez et al., 2013), and apple *MdGolS2* gene confers tolerance to water deficit when expressed in *Arabidopsis* (Falavigna et al., 2018).

Epigenetic mechanisms have been postulated to participate in the control of both, bud phenology and cold acclimation traits. In Norway spruce, the environmental temperature during embryogenesis and seed maturation affects the duration and intensity of bud dormancy and cold acclimation in the progeny, by an “epigenetic memory” process (Johnsen et al., 2005). This epigenetic mechanism has been proposed to modify the expression of certain microRNAs and genes related to bud break, such as *EBB1*, leading to different epitypes with the same genotype (Yakovlev et al., 2010, 2011; Carneros et al., 2017).

### Cell Growth Control and Phytohormone Pathways

The popular view of bud dormancy as a dynamic state of meristems moving between phases with varying depth during the low temperature period (Cooke et al., 2012) suggests that environmental and intrinsic signals are constantly interacting to determine such dormancy state, like a trade-off between cell growth and quiescence factors. For that reason, cell cycle and expansion pathways are hypothetical targets of those factors modulating dormancy induction and maintenance. In fact, Horvath et al. (2003) have brilliantly reviewed bud dormancy regulation under the perspective of cell cycle regulation and phytohormones action, substantiated on transcriptomic studies and the *Arabidopsis* model. Few more recent functional studies have provided new insights into that picture. *AINTEGUMENTALIKE1-4* (*AIL1-AIL4*) genes code for transcription factors of the AP2 family in hybrid aspen that mediate short-day dependent growth cessation (Karlberg et al., 2011). This effect on growth has been explained by direct regulation of cell cycle since AIL1 protein interacts with the promoter of *CYCD3.2*, and *AIL1* overexpression prevents down-regulation of D-type cyclins under short-day treatment. In addition, *AIL* genes are proposed to act downstream of *FT* and *AP1* genes in the photoperiodic pathway (Karlberg et al., 2011; Azeez et al., 2014), providing an interesting link of photoperiod perception with cell cycle control. More evidences of this link have been obtained from the study of cell cycle genes promoters. *Populus* plants transformed with the promoters of *Arabidopsis* cell cycle genes *CYC1* and *CDC2a* fused to a reporter gene have shown that both promoters respond to release of apical dominance by shoot decapitation, and *CYC1* promoter activity associates with daylength (Rohde et al., 1997).

A recent reappraisal of public genomic data from buds of *Arabidopsis*, grapevine, and *Populus* undergoing the growth to dormancy transition has found a common regulatory network that resembles the low energy syndrome (LES), a response triggered under carbon starvation and energy limiting conditions (Tarancón et al., 2017). LES is mediated by Sucrose Non-Fermenting-1-Related Protein Kinase (SnRK1), which ultimately results in cell division arrest and metabolic reprogramming (Martín-Fontecha et al., 2018). This idea is in remarkable agreement with the indirect theory of apical dominance postulating that stem growth inhibits axillary bud outgrowth by diverting sugars away from buds (Mason et al., 2014; Kebrom, 2017).

Also plant hormones play an important role in LES and the growth-dormancy trade-off, with gibberellins (GAs) and auxins acting as promoters of cell growth, whereas ABA associates with dormancy maintenance. GA content in *Prunus mume* changes across bud dormancy phases, in concordance with the expression of biosynthetic *GA20ox* genes (Wen et al., 2016). Moreover, application of exogenous active GA increases bud break in *Prunus mume* (Zhuang et al., 2013), and induces shoot elongation under short-days in *Salix pentandra* (Junttila and Jensen, 1988). A set of transgenic *Populus* plants with altered GA metabolism and signaling show faster growth cessation in response to short photoperiod, early bud set and delayed bud break as compared with the wild type (Zawaski et al., 2011; Zawaski and Busov, 2014). On the contrary, hybrid aspen plants with increased GA concentration by overexpression of *AtGA20ox1* continue to grow under short-day conditions (Eriksson et al., 2015). In addition to its role in apical dominance/paradormancy induction, gene expression studies associate auxin signaling with bud dormancy release and growth resumption (Anderson et al., 2005; El Kayal et al., 2011; Noriega and Pérez, 2017). On the other hand, modification of ABA signaling by overexpression and down-regulation of a poplar ortholog of *ABA INSENSITIVE 3* (*ABI3*) alters bud formation in response to short-days (Rohde et al., 2002; Ruttink et al., 2007). Interestingly, ABI3 protein interacts with FLOWERING LOCUS D 1 (FDL1), pointing to an orchestrated control of bud development by photoperiodic and ABA pathways (Tylewicz et al., 2015; Singh et al., 2017). In addition, hybrid aspen plants with a reduced ABA response by expressing the dominant allele *abi1-1* of the ABA signaling gene *ABI1* show growth cessation and form buds under short photoperiod, but remain in a non-dormant state, arguing for a specific effect of ABA on dormancy induction (Tylewicz et al., 2018). In grapevine, ABA has been postulated to affect bud dormancy development through the modulation of the expression of cell cycle genes (Vergara et al., 2017). Similarly to *ABI3* overexpressing lines, birch (*Betula pendula*) plants made insensitive to ethylene by expressing the dominant mutation *etr1-1* of the ethylene receptor *ETR1* show alterations in bud formation (Ruonala et al., 2006). In the same study, *etr1-1* plants fail to accumulate ABA in response to short-days, which suggests an interplay of both hormones in bud development mechanisms. The role of these hormones in bud dormancy pathways have been largely supported by transcriptomic studies in different species (Bai et al., 2013; Doğramaci et al., 2013; Howe et al., 2015) and metabolic profiling (Chao et al., 2016).

## Growth Resumption and Flowering

### A Molecular Calendar for Dormancy Release

Bud dormancy release integrates cumulative chilling perception into a molecular calendar mechanism that triggers growth resumption after fulfillment of the specific chilling requirements of a given species or genotype. Our current knowledge about both seasonal temperature sensing and calendar mechanisms is still scarce and fragmentary, although some common and specific elements of these regulatory circuits have been already described in different species. *FT* gene and the growth-promoting hormones GAs have been postulated as main factors leading to bud dormancy release in trees (Brunner et al., 2014; Maurya and Bhalerao, 2017; Singh et al., 2017). Exposure to seasonal low temperature leads to up-regulation of GA biosynthetic genes and down-regulation of GA catabolic genes during the dormancy-activity transition in hybrid aspen (Karlberg et al., 2010), and up-regulation of *FT1* gene in poplar (Hsu et al., 2011; Rinne et al., 2011). Furthermore, seasonal chilling induces the GA-dependent expression of 1,3-β-D-glucanase genes, involved in removal of callose sphincters on plasmodesmata and the subsequent reopening of cell-to-cell communication in meristematic cells (Rinne et al., 2001; Rinne et al., 2011). The key role of cell-to-cell communication closure in dormancy induction has been recently confirmed by studying hybrid aspen plants with altered sensitivity to ABA (Tylewicz et al., 2018). Mobile peptides such as FT and CENTRORADIALIS (CEN) are possible candidates moving through those open plasmodesmata to control cell proliferation (Rinne et al., 2011; Tylewicz et al., 2018). Poplar transgenic plants overexpressing *CEN1* gene require an extended chilling time for bud break (Mohamed et al., 2010), suggesting that *CEN1* counteracts the flowering promoting effect of *FT1* gene, and that relative levels of *FT1* and *CEN1* could determine dormancy release (Brunner et al., 2014). Similar results have been obtained in kiwifruit (Varkonyi-Gasic et al., 2013).

Besides, the *EARLY BUD-BREAK 1* (*EBB1*) gene codes for an AP2 type transcription factor that has been associated with bud break events in different species. *EBB1* has been identified as the tagged gene in a dominant mutant of poplar showing early bud break, whereas down-regulation of *EBB1* delays bud break (Yordanov et al., 2014). *EBB1* sequence and expression profile is conserved in other perennials, which suggests its positive participation in bud break across a wide range of tree species (Busov et al., 2016). In fact, Japanese pear *PpEBB* gene is up-regulated during the rapid enlargement stage in ecodormant buds prior to bud break events and induces the expression of several cyclin *PpCYCD3* genes in transient expression assays, providing a link with cell division mechanisms required for bud break and blooming (Tuan et al., 2016).

In Rosaceae species and leafy spurge (*Euphorbia esula*), *DAM* genes are also considered major chilling-dependent regulators of bud dormancy, and thus are also putative components of their respective molecular calendars. The *evg* mutant of peach, showing a non-dormant phenotype, contains a partial deletion of a tandemly repeated family of *DAM* genes (Bielenberg et al., 2008). *DAM* genes are specifically expressed in dormant vegetative and reproductive buds, and down-regulated concomitantly with dormancy release events, although several *DAM* family members show gene expression particularities (Li et al., 2009; Jiménez et al., 2010b; Yamane et al., 2011; Kitamura et al., 2016). Other MADS-box domain genes (i.e., *FLOWERING LOCUS C*-like and *SHORT VEGETATIVE PHASE*-like) have been related to chilling requirements and dormancy release in apple (*Malus* × *domestica*) and kiwifruit (*Actinidia deliciosa*) among other species (Porto et al., 2015; Wu et al., 2017a,b).

Resembling the vernalization-dependent flowering in *Arabidopsis*, epigenetic modifications including chromatin histone methylation and acetylation, DNA methylation and small RNA regulation have been postulated to mediate chilling dependent release of dormancy (Horvath et al., 2003; Ríos et al., 2014). Concomitantly with cold accumulation and gene down-regulation, the chromatin in regulatory regions of *DAM* genes in leafy spurge and peach show a decrease in trimethylation of histone H3 at lysine 4 (H3K4me3) and an increase of trimethylated H3 at lysine 27 (H3K27me3), which are modifications associated with gene repression and silencing (Horvath et al., 2010; Leida et al., 2012a). However, additional functional approaches are required in order to state a role of these chromatin marks in *DAM*-dependent regulation of dormancy release by chilling. Down-regulation of the chromodomain/helicase/DNA-binding domain (CHD3) *PICKLE*, a known antagonist of H3K27me3 modification in *Arabidopsis* (Aichinger et al., 2009), restores plasmodesmata closure and photoperiod-dependent bud dormancy in ABA response defective plants, suggesting that ABA promotes bud dormancy by repressing *PICKLE* (Tylewicz et al., 2018). Also, methylation of DNA affects chromatin structure and gene-specific expression, and thus it may potentially account for large transcriptomic rearrangements observed in developmental transitions. In effect, global and specific levels of genomic DNA cytosine methylation change during bud development in chestnut (Santamaría et al., 2009) and apple (Kumar et al., 2016), and recent functional studies reveal the important role of DNA methylation enzymes in seasonal dormancy regulation: overexpression of a chestnut *DEMETER-like* (*CsDML*) DNA demethylase accelerates photoperiodic-dependent bud formation (Conde et al., 2017b), whereas down-regulation of poplar *DEMETER-like* (*PtaDML10*) delays bud break (Conde et al., 2017a) in poplar. In sweet cherry (*Prunus avium*), specific DNA methylations and siRNAs are associated with silencing of the *DAM*-like gene *PavMADS1* during dormancy release (Rothkegel et al., 2017). Modification of transcript stability by microRNA action has been also hypothesized to participate in bud dormancy regulation. The aspen microRNA ptr-MIR169 represses the expression of *Heme Activator Protein 2* (*ptrHAP2*) in dormant buds (Potkar et al., 2013), a component of nuclear factor Y (NF-Y) complexes involved in regulation of flowering in *Arabidopsis* by modulating the epigenetic state of target genes (Hou et al., 2014), which provides a potential way for regulation of *FT*. In pear, *DAM* transcripts are targeted and degraded by miR6390 microRNA, thus contributing to *DAM* down-regulation in the bud dormancy release transition (Niu et al., 2016).

### Flowering Pathways

Flowering pathways and genes are broadly conserved between herbaceous and perennial plants, in spite of their evident phenological particularities. In perennials, a period of seasonal dormancy usually interposes between flower induction and blooming (Boes and Strauss, 1994; Kurokura et al., 2013), which forces the mutual coordination of flowering, dormancy and cold acclimation processes. Under these circumstances, pre-existing components of flowering pathways have apparently evolved to acquire new functionalities adapted to the growth of perennials in temperate climates. The proposed functions of *FT1* in flower induction and dormancy release and *FT2* in the regulation of photoperiodic growth cessation in poplar constitute a paradigmatic case of neo-functionalization after a gene duplication event in trees, in contrast to the main role of *FT* in the transition to flowering in *Arabidopsis* (Pin and Nilsson, 2012). A role for *FT* and the similar but functionally antagonist *TFL* genes in flower induction has been also postulated in other perennial species different from poplar, based on expression and transgenic studies (Kotoda and Wada, 2005; Jones et al., 2011; Ziv et al., 2014; Bai et al., 2017; Reig et al., 2017). Similarly, orthologs of *Arabidopsis* flowering genes *LEAFY* (*LFY*) and *AP1* perform a function related to flowering transition in perennial species. *LFY*-like genes from trees are preferentially expressed during flower induction and accelerate flowering when ectopically expressed in *Arabidopsis*, however no evidences of their flowering promoting effect have been observed when overexpressed in poplar (Rottmann et al., 2000). On the contrary, RNAi of *PtLFY* induces sterility and delays bud break in poplar (Klocko et al., 2016). On the other hand, a dominant negative mutation of *AP1* from *Arabidopsis* modifies the regulation of flowering related genes in poplar (Chen et al., 2015), and overexpression of *AP1*-like gene from *Salix integra* induces early flowering in haploid poplar (Yang et al., 2018). In addition to homologs of known flowering genes, miRNAs and hormone signaling pathways have been proposed to integrate developmental and environmental cues affecting flower induction (Xing et al., 2015; Guo et al., 2017).

The reproductive development in perennials is closely associated with phenology. Following flower induction, reproductive organs differentiate and continue growing until a given developmental stage is reached before the dormancy period. In peach and apricot, dormant anthers are arrested in the form of sporogenous tissue (Julian et al., 2011; Ríos et al., 2013). Then after dormancy release, pollen mother cells undergo meiosis followed by pollen development and maturation, and ovaries start to form ovules (Luna et al., 1990; Julian et al., 2011). The harmful effect of cold and other environmental stresses on microsporogenesis, leading to ploidy alterations in male gametes and sterility (De Storme and Geelen, 2014), suggests that dormancy arrest in a pre-meiosis stage may serve to ensure a proper production of male gametes under more favorable environmental conditions.

### A Peach Transcriptomic Model for Bud Studies

**FIGURE 1:**
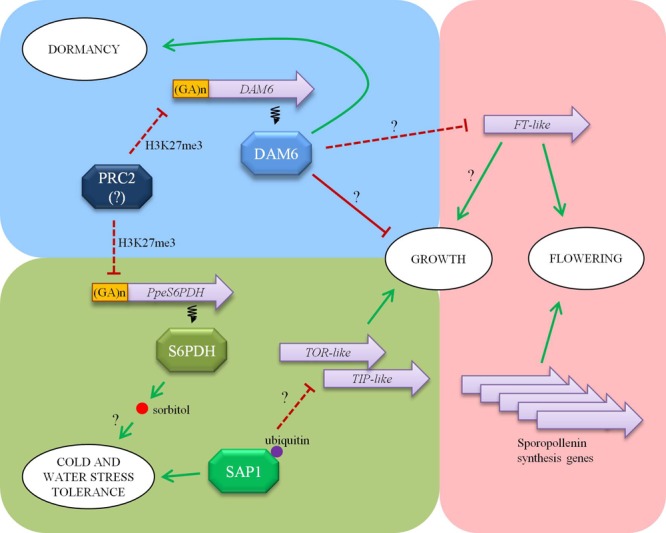
An overview of main processes converging in a flower bud of peach: dormancy (blue background), stress tolerance (green), and flowering (pink). Dormancy-Associated MADS-box 6 (DAM6) favors dormancy maintenance while repressing growth resumption and flowering. Sorbitol-6-phosphate dehydrogenase (S6PDH) enzyme synthesizes sorbitol in dormant buds, performing a putative role as cryoprotectant and compatible solute. Polycomb Repressive Complex 2 (PRC2) has been proposed to repress *DAM6* and *PpeS6PDH* genes by H3K27me3 modification of a chromatin stretch enriched in GA repeats. Stress associated protein 1 (SAP1) is a regulatory protein with ubiquitin binding ability that modulates water loss and cell growth. This last function could be partially mediated by down-regulation of cell growth regulatory (*TOR-like*) and vacuolar aquaporin (*TIP-like*) genes. *FLOWERING LOCUS T* (*FT*)-like is a main regulator of growth and flowering processes, and hypothetically integrates environmental and physiological signals. Other flowering related genes, such as the ones involved in sporopollenin biosynthesis and pollen maturation are also linked to dormancy release and growth resumption events. Arrows indicate genetic or biochemical activation while lines ending in a bar indicate repression. Transcriptional interactions are depicted with discontinuous lines. Question marks tag the relations that have not been confirmed yet.

Over the last few years, a pleiad of transcriptomic studies have provided abundant data about gene expression across bud development in white spruce (El Kayal et al., 2011), poplar (Ruttink et al., 2007), oak (Ueno et al., 2013), raspberry (Mazzitelli et al., 2007), apple (Falavigna et al., 2014), pear (Bai et al., 2013), Japanese apricot (Habu et al., 2014), peach (Jiménez et al., 2010a), *Vitis riparia* (Mathiason et al., 2009), and leafy spurge (Horvath et al., 2008) among other perennial species. In our laboratory, we have initiated a transcriptomic approach using flower buds of peach at different dormancy stages and cultivars with different chilling requirements (Leida et al., 2010). The systematic study of differentially expressed transcripts identified in this study has provided a dynamic snapshot of biological processes taking place in a flower bud across dormancy release, including regulation of dormancy release, tolerance to abiotic stresses and flower development (**Figure 1**).

Dormancy-associated genes *DAM1, 4, 5*, and *6*, belonging to the family of 6 tandemly arrayed *DAM* genes, have been found down-regulated in flower buds of peach following dormancy release, and differentially expressed in cultivars with different chilling requirements (Leida et al., 2010, 2012a). These genes share a common chromatin modification involving H3K27me3 enrichment after dormancy release (de la Fuente et al., 2015), suggesting thus a putative mechanism for gene silencing similar to the epigenetic regulation of the vernalization-responsive *FLC* gene in *Arabidopsis* (Ríos et al., 2014). H3K27me3 epigenetic mark in peach buds is associated with genomic (GA)n repeats, in concordance with the role of (GA)n binding proteins in recruiting the Polycomb repressive complex 2 (PRC2) involved in trimethylation at H3K27 in *Arabidopsis* (Xiao et al., 2017). *FT*-like gene is up-regulated in dormancy-released buds in peach, following an opposite pattern to *DAM6* (Leida et al., 2012b), which resembles down-regulation of *FT* by *DAM* genes found in other species (Hao et al., 2015), and provides a mechanism by which *DAM* genes might mediate growth and dormancy responses.

In addition to *DAM*, many other genes are differentially enriched in the H3K27me3 mark in buds undergoing dormancy release. Among them, *PpeS6PDH* codifies a sorbitol-6-phosphate dehydrogenase involved in sorbitol synthesis that is expressed in dormant buds and down-regulated in dormancy released buds concomitantly with an increase in H3K27me3 modification (Lloret et al., 2017b). This correlates with sorbitol accumulation in dormant buds, and has prompted us to postulate a role of *PpeS6PDH* and sorbitol in protection against cold and hydric stresses (**Figure 1**). In that case, it would mean that bud dormancy and stress tolerance share common regulatory epigenetic mechanisms, which are apparently independent from the well known cold acclimation pathway since the H3K27me3 mark is concurrently established in *DAM6* and *PpeS6PDH*, linked to the dormancy stage instead of the environmental temperature.

The study of gene expression in flower buds also has served to identify a *stress associated protein* (*SAP*)-like gene (*PpSAP1*) expressed in dormant buds and down-regulated concomitantly with dormancy release (Lloret et al., 2017a). SAP-like proteins containing Zn-finger domains A20 and AN1 have been found to regulate the abiotic stress response in different especies (Giri et al., 2013), most likely by an ubiquitin-related mechanism. The ectopic expression of *PpSAP1* in plum alters water loss and leaf morphology, suggesting that has a dual role in stress tolerance and cell growth (**Figure 1**). This effect on cell growth could be mediated by down-regulation of *target of rapamycin* (*TOR*)-like, a key regulator of cell growth and metabolism in eukaryotic cells, and *tonoplast intrinsic protein* (*TIP*)-like, a tonoplast aquaporin affecting water permeability and cell turgor. This makes tempting to speculate that *PpSAP1* might coordinate both growth inhibition and stress tolerance in dormant buds.

Finally, we have identified several genes transiently up-regulated after dormancy release that are specifically expressed in anthers (Ríos et al., 2013). Among these genes we have found some orthologs of *Arabidopsis* genes involved in synthesis of sporopollenin (a pollen cell wall component) and pollen maturation, which provides a molecular framework to characterize the mechanisms acting in growth resumption of reproductive organs and microsporogenesis initiated shortly after dormancy release. Altogether, the data obtained in these studies has contributed to outline a landscape of concerted cross-regulation of dormancy, stress response and flowering processes converging in a flower bud.

## Perspectives

In trees from temperate climates, dormancy is a process required for survival during winter, but the molecular pathways that regulate it are poorly known. A better understanding of the molecular bases of bud dormancy will strongly facilitate plant breeding tasks aimed at assessing the potential for environmental adaptability of particular genotypes, and studies led to evaluate the impact of climate change on crop yields. In our opinion, as a result of our experience in the peach model, this will be better achieved by approaches involving the coordinate study of dormancy, flowering and stress pathways in buds, hence providing an added value to the molecular characterization of these processes separately. Moreover, integrating approaches will help to identify common regulatory mechanisms, thus contributing to decipher the time and spatial fitting of these processes.

According to the profuse literature mentioned in this review, numerous environmental inputs transmitting temperature and light data are found in different nodes of regulatory networks, ensuring a precise tuning of phenological transitions. On the other side, quantitative and delayed responses seem to be mediated by the epigenetic machinery, which employs common chromatin labels for dormancy release, stress acclimation and flowering induction in different species. In our opinion, epigenetic modifiers will become central to most molecular dormancy studies in the immediate future, with an increasingly important impact on development and environmental adaptation fields.

## Author Contributions

AL, MB, and GR wrote the manuscript. All the authors read and approved the final version of the manuscript.

## Conflict of Interest Statement

The authors declare that the research was conducted in the absence of any commercial or financial relationships that could be construed as a potential conflict of interest.
